# First Evaluation of Field Evolved Resistance to Commonly Used Insecticides in House Fly Populations from Saudi Arabian Dairy Farms

**DOI:** 10.3390/insects12121120

**Published:** 2021-12-14

**Authors:** Abdulwahab M. Hafez

**Affiliations:** Pesticides and Environmental Toxicology Laboratory, Department of Plant Protection, College of Food and Agriculture Sciences, King Saud University, P.O. Box 2460, Riyadh 11451, Saudi Arabia; hafez@ksu.edu.sa; Tel.: +966-566444577

**Keywords:** integrated vector management, toxicity, public health insecticides, *Musca domestica*, *Muscidae*, vector borne diseases

## Abstract

**Simple Summary:**

The house fly is one of the major carriers of several diseases that affect humans and animals. Insecticides are often used as a rapid method to control them. In this study, eight commonly used insecticides were tested against five populations of house flies collected from dairies around Riyadh, Saudi Arabia. The aim was to evaluate how toxic the insecticides were, and to find out whether the flies showed any sign of resistance against insecticides. In the tested pyrethroid insecticides, there was no or only moderate resistance in adults of both sexes compared to a known susceptible strain. In the tested organophosphate insecticides, there was low to moderate resistance in adults of both sexes compared to the susceptible strain. This study also evaluated “median lethal times” for the tested insecticides (how long a certain dose takes to kill half the exposed population), with results available for all eight insecticides: alpha-cypermethrin, deltamethrin, bifenthrin, cypermethrin, cyfluthrin, fenitrothion, chlorpyrifos, and malathion. The results of this study will be helpful for people whose job it is to plan effective house fly control programs in Saudi Arabia.

**Abstract:**

The house fly, *Musca domestica* L. (Diptera: Muscidae), is one of the major vectors of several pathogens that affect humans and animals. We evaluated the toxicity of eight insecticides commonly used for house fly control using five field populations collected from dairies in Riyadh, Saudi Arabia. Among the five tested pyrethroids, non to moderate resistance was found in adults of both sexes compared to a susceptible strain. Resistance ratios ranged from 0.5- to 7-fold for alpha-cypermethrin, 2- to 21-fold for deltamethrin, 4- to 19-fold for bifenthrin, 1- to 9-fold for cyfluthrin, and 1- to 8-fold for cypermethrin. Among the three tested organophosphates, low to moderate resistance was found among adult flies compared to the susceptible strain, and the resistance ratios ranged from 4- to 27-fold for fenitrothion, 2- to 14-fold for chlorpyrifos, and 3- to 12-fold for malathion. The median lethal times for the tested insecticides were 3–33 h for alpha-cypermethrin, 3–24 h for deltamethrin, 5–59 h for bifenthrin, 1–7 h for cypermethrin, 0.3–7 h for cyfluthrin, 6–36 h for fenitrothion, 2–21 h for chlorpyrifos, and 3–34 h for malathion. This study presents baseline data pertaining to registered public health insecticides, and the results will assist future studies monitoring insecticide resistance, and the planning of effective integrated vector management programs.

## 1. Introduction

The domestic house fly, *Musca domestica* L. (Diptera: Muscidae), is a major insect pest in rural and urban areas worldwide [1,2,3,4]. It is a nuisance, causes food spoilage, serves as a carrier of numerous pathogens causing diseases in humans and livestock [5,6], and has been shown to transmit more than 200 human and animal pathogens associated with fatal diseases [7].

A variety of organophosphate and pyrethroid insecticides have been recommended to manage various insect pests, including the house fly, worldwide. However, over the past few decades, over-reliance on synthetic insecticides has resulted in the house fly developing resistance to these two classes of insecticide, increasing the challenges for insect pest management [8,9,10,11]. Overuse of insecticides has also resulted in environmental pollution, increased the cost of preventive control, and caused destruction of non-target organisms [12,13]. These issues emphasize the necessity to employ an integrated pest management program against insect pests, including the house fly [14,15,16]. To overcome the development of resistance, excessive applications of insecticides at increasing dose rates and more frequent intervals have been used, but these practices have escalated the problem and rendered the control of house fly even more difficult all over the world, particularly in areas where most suitable insecticides have lost their efficacy [17].

Studies monitoring resistance of insecticides constitute one of the most important strategic components of insect pest management. They can identify resistance early and constitute a critical part of the decision-making process in pest control programs [18,19,20]. Monitoring of insecticide resistance in the house fly has been reported from various countries, including Pakistan [8,9], the USA [3,10], and China [11,21]. To our knowledge, there are no reports of resistance monitoring for the most commonly used insecticides in the control of house fly populations in dairies in Saudi Arabia. Therefore, our aim was to evaluate the toxicity and resistance of eight commonly used insecticides (five pyrethroids and three organophosphates) in populations of house flies in dairies around Riyadh, Saudi Arabia.

## 2. Materials and Methods

### 2.1. Collection and Rearing of House Fly Populations

Populations were collected separately from five dairy farms located in Dirab (24.49° N, 46.60° E), Al-Masanie (24.57° N, 46.72° E), Al-Uraija (24.62° N, 46.66° E), Al-Washlah (24.39° N, 46.66° E), and Al-Muzahmiya (24.47° N, 46.23° E) in Riyadh, Saudi Arabia. Approximately 150–200 house fly adults of mixed sex were captured using 12-liter plastic jars from each dairy farm separately. The trapped flies were provided with dry sugar–milk mixture, and then transported to the Pesticide and Environmental Toxicology Laboratory at King Saud University, Riyadh, Saudi Arabia on the same day of collection. Each population was transferred into separate transparent cages (40 × 40 × 40 cm) to obtain F1 progeny. An adult diet (sugar + powdered milk at a 1:1 ratio by weight) and distilled water-soaked cotton wicks placed in glass petri dishes (5 cm in diameter) were provided to the adults. Every 2 days, fresh food was provided. The cotton wicks were moistened daily and replaced every 2 days. After 2 days in the laboratory, an artificial oviposition medium and a diet for newly hatched larvae (consisting of a paste of wheat bran, yeast, sugar, and milk at a ratio of 20:5:1.5:1.5 g, mixed with 70 mL distilled water, in 400 mL plastic cups, 2 cups/cage) [16] were placed in the adult cages. The plastic cups containing eggs were removed from the adult cages daily and covered with a muslin cloth to prevent larvae escaping. When the larvae had consumed the diet in the plastic cups, they were transferred into glass beakers containing fresh larval medium until the pupal stage. The emerged adults were transferred into rearing cages (40 × 40 × 40 cm) for mating and continuity of the life cycle. All populations were well maintained under constant conditions of 27 °C ± 2 °C, 60–70% relative humidity, and 12:12 h (light:dark) photoperiod.

The susceptible strain, used as a reference for other populations, was originally obtained from the Laboratory of Public Health Pests, Jeddah Municipality, Saudi Arabia, in 2010, and had been maintained since then under the abovementioned protocol with no exposure to any kind of chemicals.

### 2.2. Insecticides

A total of eight commercial-grade formulated pyrethroid and organophosphate insecticides were used for bioassays. The five tested pyrethroids were cypermethrin (Montothrin 10EC, Montajat Veterinary Tool Products, Dammam, Saudi Arabia), bifenthrin (Biflex 8SC, FMC, Pelt, Belgium), deltamethrin (K-Othrine 25SC, Bayer Crop Sciences, Valbonne, France), cyfluthrin (Solfac 050EW, Bayer Crop Sciences, Leverkusen, Germany), and alpha-cypermethrin (Alphaquest 100EC, Astrachem, Riyadh, Saudi Arabia). The three tested organophosphates were fenitrothion (Fentox 500EC, Pioneers Chemicals Factory Co., Riyadh, Saudi Arabia), chlorpyrifos (Chlorfet 48EC, Masani Chemicals, Amman, Jordan), and malathion (Delthion 570EC, Saudi Delta Company, Riyadh, Saudi Arabia).

### 2.3. Adult Diet Incorporation Bioassay

The toxicities to adult male and female flies of the eight insecticides were separately evaluated using feeding bioassays following the method of Abbas et al. [8]. Adult flies were anesthetized using diethyl ether (BDH Laboratory Supplies, Lutterworth, United Kingdom) for 30 s, and sexes were separated based on space between compound eyes (greater in the female than in the male) [16]. Five concentrations of each insecticide causing mortality between 0% and 100% were prepared in a 20% sugar solution through serial dilution, with three replicates for each concentration in each bioassay. In total, 10 sex-separated adults in each replicate, 30 sex-separated adults at each concentration, and 150 sex-separated adults were used in each bioassay, with 30 adult flies of each sex (10 adults/replicate) used in the control treatment. The adult flies were transferred into 1.8-liter aerated plastic jars and covered with a muslin cloth to prevent escape. A 3 cm cotton wick was soaked with a solution of each insecticide at each concentration and placed in a 9 cm diameter petri dish, and the dishes were then placed into each jar for adult feeding. In the control treatment, adult flies were exposed to a 20% sugar solution only. The cotton wicks were moistened daily with water to prevent drying. All bioassays were conducted under the abovementioned conditions. Mortality was recorded after 48 h of exposure to determine median lethal concentration (LC_50_) of the insecticides due to fast action [8]. The highest concentration (256 part per million “ppm” for Alpha-cypermethrin and 2048 ppm for the rest) used for bioassay was also used to determine the median lethal time (LT_50_), with mortality recorded after 1, 12, 24, and 48 h of exposure.

### 2.4. Data Analysis

The bioassay data were analyzed using POLO Plus software version 1 [22] to determine the values for LC_50_ and LT_50_. The formula of Abbott [23] was considered to correct the mortalities of each bioassay using the mortality of its control treatment. However, in current study, all control treatments showed zero mortalities. The LC_50_ and LT_50_ values were considered significantly different if their 95% fiducial limits (FL) did not overlap [24]. The resistance ratio (RR) was calculated as follows: RR = LC_50_ of the field population/LC_50_ of the susceptible strain. The resistance levels of the different insecticides were classified using the scale described by Torres-Vila et al. [25]: RR < 2 (no resistance), RR = 2–10 (low resistance), RR = 11–30 (moderate resistance), RR = 31–100 (high resistance), and RR > 100 (very high resistance).

## 3. Results

### 3.1. Resistance to Pyrethroids

Resistance to pyrethroids was absent or moderate in female house flies from all five dairy populations compared to susceptible females. Female flies from Al-Masanie were the most resistant to deltamethrin (13-fold) and bifenthrin (12-fold). Females from other locations showed low resistance to the tested pyrethroids (2- to 10-fold), except those from Al-Muzahmiya which showed no resistance to cypermethrin (1-fold). RR values ranged from 2 to 4 for alpha-cypermethrin, 3–13 for deltamethrin, 4–2 for bifenthrin, 3–9 for cyfluthrin, and 1–8 for cypermethrin (Table 1).

Non to moderate resistance against pyrethroids was also found in male house flies from the dairy populations compared to susceptible males. Male flies from Al-Masanie were the most resistant to deltamethrin (21-fold), whereas males from Dirab and Al-Washlah were the most resistant to bifenthrin (13- and 19-fold, respectively). RR values ranged from 0.5 to 7 for alpha-cypermethrin, 2–21 for deltamethrin, 6–19 for bifenthrin, 1–5 for cyfluthrin, and 1–4 for cypermethrin (Table 2).

### 3.2. Resistance to Organophosphates

Low to moderate resistance against organophosphates was observed in female house flies from the dairy populations compared to susceptible females. Female flies from Al-Muzahmiya were the most resistant to chlorpyrifos (14-fold) and fenitrothion (27-fold), whereas those from Dirab were the most resistant to fenitrothion (23-fold). RR values ranged from 7 to 27 for fenitrothion, 2–14 for chlorpyrifos, and 3–9 for malathion (Table 3).

Low to moderate resistance against organophosphates was also found in male house flies from the dairy populations compared to susceptible males. Male flies from Al-Washlah were the most resistant to fenitrothion (15-fold) and malathion (12-fold), whereas male flies from Al-Uraija were the most resistant to chlorpyrifos (14-fold). RR values ranged from 4 to 15 for fenitrothion, 5–14 for chlorpyrifos, and 3–12 for malathion (Table 4). 

### 3.3. LT_50_ of Pyrethroids and Organophosphates

The LT_50_ values for male house flies were 3–33 h for alpha-cypermethrin, 3–22 h for deltamethrin, 8–59 h for bifenthrin, 1–7 h for cypermethrin, 0.3–1 h for cyfluthrin, 6–16 h for fenitrothion, 2–11 h for chlorpyrifos, and 3–18 h for malathion. For alpha-cypermethrin, the LT_50_ values against Al-Uraija and Al-Masanie populations were significantly higher than that observed in all other tested populations (no overlapping 95% FL). While, the LT_50_ value against Al-Muzahmiya population was significantly lower than that observed in all other tested populations, except for Al-Washlah population. For deltamethrin, the LT_50_ value against Al-Masanie population was significantly higher than that observed in all other tested populations. For bifenthrin, the LT_50_ value against Al-Washlah population was significantly higher than that observed in all other tested populations, except for Al-Muzahmiya population. However, this finding may be considered not fully reliable due to the high degree of variation in Al-Washlah population 95% fiducial limits. For cypermethrin, the only significant difference in the LT_50_ values was detected between Al-Uraija (higher) and Al-Muzahmiya (lower) populations. For cyfluthrin, no significant differences were detected in the LT_50_ values among all tested populations (overlapped 95% FL). For fenitrothion, the only significant difference in the LT_50_ values was detected between Al-Washlah (higher) and Al-Uraija (lower) populations. For chlorpyrifos, the LT_50_ values against Dirab and Al-Uraija populations were significantly higher than that observed in all other tested populations. For malathion, the significant highest LT_50_ value was detected against Al-Washlah population (except for Al-Uraija population) and the significant lowest LT_50_ value was detected against Al-Muzahmiya population (except for Al-Masanie population) (Table 5).

The LT_50_ values for female house flies were 3–30 h for alpha-cypermethrin, 4–24 h for deltamethrin, 5–49 h for bifenthrin, 1–4 h for cypermethrin, 2–7 h for cyfluthrin, 14–36 h for fenitrothion, 3–21 h for chlorpyrifos, and 8–34 h for malathion. No significant differences were found in the LT_50_ values of cypermethrin, cyfluthrin, and fenitrothion among all tested populations (overlapped 95% FL). For alpha-cypermethrin, the significant highest LT_50_ value was detected against Al-Uraija population (except for Al-Masanie and Al-Washlah populations) and the significant lowest LT_50_ value was detected against Al-Muzahmiya population (except for Dirab and Al-Masanie populations). For deltamethrin, the LT_50_ value against Al-Washlah population was significantly higher than that observed in all other tested populations, except for Al-Uraija population. For bifenthrin, the LT_50_ value against Dirab population was significantly lower than that observed in all other tested populations, except for Al-Washlah population. However, this finding may be considered not fully reliable due to the high degree of variation in Al-Masanie population 95% fiducial limits. For chlorpyrifos, the significant highest LT_50_ value was detected against Al-Uraija population (except for Al-Muzahmiya population) and the LT_50_ value against Al-Washlah population was significantly lower than that observed in all other tested populations. For malathion, the significant highest LT_50_ value was detected against Al-Muzahmiya population (except for Al-Washlah population) and the significant lowest LT_50_ value was detected against Dirab population (except for Al-Uraija population) (Table 6).

## 4. Discussion

Synthetic chemicals have been recommended for the management various pests, including house flies, in Saudi Arabia [26]. Genetically based decline in susceptibility to an insecticide in a field population is known as field evolved resistance [27]. Evaluating the toxicity of and resistance to different synthetic chemicals is a key aspect in selection of the most effective compound to manage disease vectors. Therefore, the present study was performed to assess the resistance of house flies from five dairy facilities to five pyrethroid (alpha-cypermethrin, deltamethrin, bifenthrin, cyfluthrin, and cypermethrin) and three organophosphate (fenitrothion, chlorpyrifos, and malathion) insecticides. The results of the present study revealed <10-fold field evolved resistance in female house flies to alpha-cypermethrin, cyfluthrin, cypermethrin, and malathion in all five populations, deltamethrin in three populations, bifenthrin in four populations, fenitrothion in one population, and chlorpyrifos in two populations. However, male house flies showed ≤10-fold field evolved resistance to alpha-cypermethrin, cyfluthrin, and cypermethrin in all five populations, deltamethrin in four populations, bifenthrin in three populations, fenitrothion and chlorpyrifos in two populations, and malathion in four populations. These populations showed low levels of field evolved resistance while the remaining populations showed moderate levels of field evolved resistance to the tested insecticides. Previously, high levels of pyrethroid and organophosphate insecticide resistance have been documented in house flies from various parts of the world, including Turkey [28], Pakistan [8,16,29,30], the USA [3,10], and China [11].

Pyrethroids, which are sodium channel modulators, have been used to manage various disease vectors worldwide [8,9,31]. In the present study, no to moderate resistance was observed in male and female house flies from different dairy facilities against the tested pyrethroids. Female flies in Al-Masanie showed moderate field evolved resistance to deltamethrin (13-fold) and bifenthrin (12-fold). Male flies in Al-Masanie showed moderate field evolved resistance to deltamethrin (21-fold), while male flies in Dirab (13-fold) and Al-Washlah (19-fold) showed moderate resistance to bifenthrin. Resistance of insect vectors to pyrethroids has been extensively investigated in different countries, including in house flies [3,8,9,29], *Aedes aegypti* (L.) and *Aedes albopictus* (Skuse) [31], *Culex quinquefasciatus* (Say) [20], *Culex pipiens* [32], *Anopheles gambiae* (Giles) [33], and *Anopheles stephensi* (Liston) [34].

Organophosphates, which are acetylcholinesterase inhibitors, are the most commonly used insecticides across the world to manage several pests, including the house fly [8,35]. However, resistance to organophosphates has been documented in the house fly [8,28,30], *Cx. quinquefasciatus* [20], *Ae. albopictus* [36], *Tuta absoluta* (Meyrick) [18], and *Phenacoccus solenopsis* (Tinsley) [37], with varying ranges of resistance being reported. Among the tested organophosphates in the current study, low to moderate resistances to fenitrothion, chlorpyrifos, and malathion were detected in the house fly populations from the tested regions. Resistance levels can depend upon the use of insecticides at dairy facilities [3,8]. In the present study, non to low levels of resistance to pyrethroids and organophosphates in most populations suggests that these insecticides are still effective in Saudi Arabian dairy facilities for the management of house flies. However, with some populations approaching moderate resistance, unwise use of these insecticides may lead to the development of resistance in the future. Therefore, a strategic program should be developed for the management of house flies in order to delay the development of resistance and to sustain the efficacy of these insecticides.

In conclusion, the house fly populations that were collected from different dairy farms in Riyadh, Saudi Arabia, exhibited no to moderate resistance to pyrethroids and low to moderate resistance to organophosphates. Therefore, these insecticides should be used carefully with periodic monitoring to detect any further increases in resistance. The limited use of insecticides to which resistance has developed, the use of mixtures of insecticides with unrelated mechanisms of action, and appropriate cultural practices may help in managing house fly insecticide resistance. Insect growth regulators, biopesticides, as well as appropriate cultural practices, should be included in integrated vector management programs designed to control house fly populations, to reduce the selection pressure on the commonly used insecticides [26,38,39,40]. The findings of this study can serve as a reference in future monitoring efforts of house fly insecticide susceptibility.

## Figures and Tables

**Table 1:** Toxicity of pyrethroids in adult female house flies from different dairy farms.

Insecticide	Population	Year	^1^ N	Slope ± SE	χ^2^	*p*	^2^ LC_50_	^3^ FL (95%)	^4^ RR
Alpha-cypermethrin	Susceptible	-	180	1.4 ± 0.3	0.8	0.8	42	29–61	1
	Dirab	2019	180	2.4 ± 0.4	6.8	0.1	90	46–230	2
	Al-Masanie	2019	180	0.9 ± 0.3	0.6	0.9	160	93–557	4
	Al-Uraija	2019	180	1.2 ± 0.3	1.4	0.7	136	88–289	3
	Al-Washlah	2019	180	2.5 ± 0.5	2.3	0.5	89	71–114	2
	Al-Muzahmiya	2019	180	1.8 ± 0.3	2.6	0.5	86	64–119	2
Deltamethrin	Susceptible	-	180	1.3 ± 0.3	0.6	0.4	71	48–116	1
	Dirab	2019	180	0.9 ± 0.3	0.1	1.0	205	59–354	3
	Al-Masanie	2019	180	1.8 ± 0.3	1.8	0.6	889	667–1283	13
	Al-Uraija	2019	180	1.1 ± 0.3	0.7	0.9	322	170–497	5
	Al-Washlah	2019	180	2.4 ± 0.3	7.5	0.1	698	371–1698	10
	Al-Muzahmiya	2019	180	1.4 ± 0.3	1.3	0.7	398	262–566	6
Bifenthrin	Susceptible	-	180	1.0 ± 0.3	0.2	0.7	139	87–265	1
	Dirab	2019	180	1.5 ± 0.3	4.9	0.2	975	697–1570	7
	Al-Masanie	2019	180	1.1 ± 0.3	0.4	0.9	1638	984–4944	12
	Al-Uraija	2019	180	1.0 ± 0.3	0.7	0.9	651	340–3949	5
	Al-Washlah	2019	180	1.1 ± 0.3	0.7	0.9	552	342–926	4
	Al-Muzahmiya	2019	180	1.6 ± 0.3	2.7	0.4	1025	737–1643	7
Cyfluthrin	Susceptible	-	180	1.6 ± 0.3	1.0	0.5	123	89–177	1
	Dirab	2019	180	1.7 ± 0.3	5.7	0.1	580	428–800	5
	Al-Masanie	2019	180	1.2 ± 0.3	2.9	0.4	473	304–722	4
	Al-Uraija	2019	180	1.5 ± 0.3	5.1	0.2	490	345–690	4
	Al-Washlah	2019	180	0.8 ± 0.3	2.2	0.5	1107	605–4966	9
	Al-Muzahmiya	2019	180	0.8 ± 0.3	0.8	0.8	304	90–558	3
Cypermethrin	Susceptible	-	180	1.3 ± 0.3	0.7	0.5	70	42–104	1
	Dirab	2019	180	1.1 ± 0.3	2.0	0.6	211	82–341	3
	Al-Masanie	2019	180	2.0 ± 0.3	1.3	0.7	406	303–530	6
	Al-Uraija	2019	180	1.3 ± 0.3	0.5	0.9	404	257–591	6
	Al-Washlah	2019	180	1.3 ± 0.3	0.7	0.9	571	380–885	8
	Al-Muzahmiya	2019	180	1.1 ± 0.3	0.2	1.0	80	10–162	1

^1^ Number of tested adult females. ^2^ Median lethal concentration (ppm). ^3^ Fiducial limits. ^4^ Resistance ratio. Degrees of freedom = 3.

**Table 2:** Toxicity of pyrethroids in adult male house flies from different dairy farms.

Insecticide	Population	Year	^1^ N	Slope ± SE	χ^2^	*p*	^2^ LC_50_	^3^ FL (95%)	^4^ RR
Alpha-cypermethrin	Susceptible	-	180	2.2 ± 0.3	3.6	0.9	35	19–56	1
	Dirab	2019	180	2.2 ± 0.3	6.6	0.1	82	40–120	2
	Al-Masanie	2019	180	1.2 ± 0.3	4.5	0.2	241	146–709	7
	Al-Uraija	2019	180	0.8 ± 0.3	0.1	0.9	74	39–162	2
	Al-Washlah	2019	180	3.3 ± 0.4	2.6	0.5	59	49–71	2
	Al-Muzahmiya	2019	180	1.0 ± 0.3	2.1	0.5	19	5–33	0.5
Deltamethrin	Susceptible	-	180	1.3 ± 0.3	0.5	0.2	47	31–69	1
	Dirab	2019	180	0.8 ± 0.3	0.2	1.0	114	9–236	2
	Al-Masanie	2019	180	1.6 ± 0.3	1.6	0.6	983	704–1579	21
	Al-Uraija	2019	180	1.3 ± 0.3	0.3	0.9	133	48–215	3
	Al-Washlah	2019	180	1.3 ± 0.3	0.8	0.8	299	168–443	6
	Al-Muzahmiya	2019	180	1.3 ± 0.3	1.3	0.7	97	25–173	2
Bifenthrin	Susceptible	-	180	1.2 ± 0.3	0.2	0.6	86	52–133	1
	Dirab	2019	180	0.9 ± 0.3	4.1	0.3	1083	600–4376	13
	Al-Masanie	2019	180	1.7 ± 0.3	2.3	0.5	470	339–643	6
	Al-Uraija	2019	180	1.7 ± 0.4	2.3	0.5	510	343–1085	6
	Al-Washlah	2019	180	0.7 ± 0.3	0.3	1.0	1591	786–2088	19
	Al-Muzahmiya	2019	180	1.3 ± 0.3	0.8	0.8	829	568–1401	10
Cyfluthrin	Susceptible	-	180	1.6 ± 0.3	3.1	0.6	85	43–154	1
	Dirab	2019	180	1.1 ± 0.3	5.2	0.2	432	247–695	5
	Al-Masanie	2019	180	1.4 ± 0.3	1.2	0.8	345	215–496	4
	Al-Uraija	2019	180	1.6 ± 0.3	0.3	1.0	358	245–489	4
	Al-Washlah	2019	180	1.9 ± 0.3	4.9	0.2	355	244–672	4
	Al-Muzahmiya	2019	180	1.3 ± 0.3	0.3	1.0	121	38–204	1
Cypermethrin	Susceptible	-	180	1.6 ± 0.3	1.9	0.1	53	34–73	1
	Dirab	2019	180	0.9 ± 0.3	0.2	1.0	74	4–166	1
	Al-Masanie	2019	180	0.9 ± 0.3	0.2	1.0	72	4–161	1
	Al-Uraija	2019	180	1.5 ± 0.3	1.3	0.7	201	108–292	4
	Al-Washlah	2019	180	0.8 ± 0.3	0.9	0.8	175	28–329	3
	Al-Muzahmiya	2019	180	2.9 ± 0.6	1.3	0.7	122	71–162	2

^1^ Number of tested adult males. ^2^ Median lethal concentration (ppm). ^3^ Fiducial limits. ^4^ Resistance ratio. Degrees of freedom = 3.

**Table 3:** Toxicity of organophosphates in adult female house flies from different dairy farms.

Insecticide	Population	Year	^1^ N	Slope ± SE	χ^2^	*p*	^2^ LC_50_	^3^ FL (95%)	^4^ RR
Fenitrothion	Susceptible	-	180	1.0 ± 0.3	1.8	0.6	37	19–61	1
	Dirab	2019	180	1.3 ± 0.3	2.0	0.6	849	587–1418	23
	Al-Masanie	2019	180	2.0 ± 0.3	6.6	0.1	548	241–1386	15
	Al-Uraija	2019	180	1.5 ± 0.3	0.7	0.9	410	279–575	11
	Al-Washlah	2019	180	1.1 ± 0.3	0.9	0.8	241	104–381	7
	Al-Muzahmiya	2019	180	1.9 ± 0.3	1.7	0.6	990	749–1425	27
Chlorpyrifos	Susceptible	-	180	1.8 ± 0.3	3.9	0.3	26	9–46	1
	Dirab	2019	180	1.1 ± 0.3	1.6	0.7	302	146–475	12
	Al-Masanie	2019	180	1.2 ± 0.3	0.7	0.9	50	9–106	2
	Al-Uraija	2019	180	1.1 ± 0.3	0.4	0.9	347	189–537	13
	Al-Washlah	2019	180	1.7 ± 0.4	1.3	0.7	120	52–145	5
	Al-Muzahmiya	2019	180	1.5 ± 0.3	0.4	0.7	352	231–494	14
Malathion	Susceptible	-	180	2.4 ± 0.3	0.5	0.1	79	52–144	1
	Dirab	2019	180	1.5 ± 0.3	0.9	0.8	267	160–380	3
	Al-Masanie	2019	180	1.2 ± 0.3	0.9	0.8	266	141–399	3
	Al-Uraija	2019	180	1.5 ± 0.3	2.7	0.4	375	249–525	5
	Al-Washlah	2019	180	1.9 ± 0.3	1.3	0.7	736	555–1023	9
	Al-Muzahmiya	2019	180	1.3 ± 0.3	5.1	0.2	680	468–1071	9

^1^ Number of tested adult females. ^2^ Median lethal concentration (ppm). ^3^ Fiducial limits. ^4^ Resistance ratio. Degrees of freedom = 3.

**Table 4:** Toxicity of organophosphates in adult male house flies from different dairy farms.

Insecticide	Population	Year	^1^ N	Slope ± SE	χ^2^	*p*	^2^ LC_50_	^3^ FL (95%)	^4^ RR
Fenitrothion	Susceptible	-	180	1.9 ± 0.3	4.6	0.2	32	13–60	1
	Dirab	2019	180	1.9 ± 0.3	4.4	0.2	280	104–490	9
	Al-Masanie	2019	180	2.3 ± 0.3	2.8	0.4	421	325–536	13
	Al-Uraija	2019	180	1.2 ± 0.3	1.6	0.7	139	44–234	4
	Al-Washlah	2019	180	1.1 ± 0.2	1.8	0.6	466	322–659	15
	Al-Muzahmiya	2019	180	3.3 ± 0.4	6.6	0.1	444	255–799	14
Chlorpyrifos	Susceptible	-	180	1.7 ± 0.3	5.6	0.1	18	10–25	1
	Dirab	2019	180	1.7 ± 0.3	1.3	0.7	195	115–272	11
	Al-Masanie	2019	180	1.6 ± 0.3	1.4	0.7	127	56–192	7
	Al-Uraija	2019	180	1.9 ± 0.3	5.4	0.2	259	159–486	14
	Al-Washlah	2019	180	3.6 ± 0.4	0.2	0.9	93	32–125	5
	Al-Muzahmiya	2019	180	1.6 ± 0.3	2.9	0.4	236	141–332	13
Malathion	Susceptible	-	180	1.6 ± 0.3	0.4	0.9	46	33–65	1
	Dirab	2019	180	2.1 ± 0.3	1.2	0.8	219	149–290	5
	Al-Masanie	2019	180	2.1 ± 0.4	0.3	0.9	121	61–173	3
	Al-Uraija	2019	180	1.3 ± 0.3	0.3	0.9	157	65–246	3
	Al-Washlah	2019	180	1.5 ± 0.3	2.0	0.6	542	385–772	12
	Al-Muzahmiya	2019	180	2.1 ± 0.3	2.8	0.4	385	292–495	8

^1^ Number of tested adult males. ^2^ Median lethal concentration (ppm). ^3^ Fiducial limits. ^4^ Resistance ratio. Degrees of freedom = 3.

**Table 5:** Median lethal time (LT_50_) of pyrethroids and organophosphates in male house flies.

Population	Conc. _ppm_	^1^ LT_50_ (h)	^2^ FL (95%)	Slope (SE)	Conc. _ppm_	^1^ LT_50_ (h)	^2^ FL (95%)	Slope (SE)
		Alpha-cypermethrin				Deltamethrin		
Dirab	256	14	12–17 b	5.8 (1.2)	2048	3	1–4 c	1.2 (0.2)
Al-Masanie	256	29	19–52 a	1.4 (0.3)	2048	22	15–33 a	1.7 (0.4)
Al-Uraija	256	33	21–68 a	1.3 (0.3)	2048	5	3–8 bc	1.3 (0.2)
Al-Washlah	256	9	6–13 bc	2.7 (0.5)	2048	9	6–14 b	1.1 (0.2)
Al-Muzahmiya	256	3	1–7 c	2.0 (0.7)	2048	3	1–6 bc	1.1 (0.2)
		Bifenthrin				Cypermethrin		
Dirab	2048	11	6–18 b	1.2 (0.2)	2048	3	1–6 ab	1.0 (0.2)
Al-Masanie	2048	8	5–13 b	1.3 (0.2)	2048	1	0–3 ab	0.8 (0.2)
Al-Uraija	-	-	-	-	2048	7	3–13 a	0.9 (0.2)
Al-Washlah	2048	59	28–461 a	0.8 (0.2)	2048	3	0–7 ab	0.7 (0.2)
Al-Muzahmiya	2048	17	10–29 ab	1.2 (0.2)	2048	1	0–2 b	1.1 (0.2)
		Cyfluthrin				Fenitrothion		
Dirab	2048	1	0–3 a	0.8 (0.2)	2048	14	9–17 ab	3.8 (0.8)
Al-Masanie	2048	0.3	0–2 a	0.4 (0.2)	2048	13	8–17 ab	2.3 (0.5)
Al-Uraija	2048	0.4	0–2 a	0.6 (0.2)	2048	6	3–10 b	1.3 (0.2)
Al-Washlah	2048	0.6	0–2 a	0.8 (0.2)	2048	16	11–20 a	2.5 (0.6)
Al-Muzahmiya	2048	1	0–2 a	1.0 (0.2)	2048	13	9–16 ab	3.5 (0.8)
		Chlorpyrifos				Malathion		
Dirab	2048	9	6–12 a	2.6 (0.5)	-	-	-	-
Al-Masanie	-	-	-	-	2048	8	5–11 bc	2.6 (0.5)
Al-Uraija	2048	11	7–15 a	2.6 (0.5)	2048	12	7–15 ab	3.0 (0.7)
Al-Washlah	2048	2	1–2 b	2.2 (0.4)	2048	18	12–23 a	2.4 (0.5)
Al-Muzahmiya	2048	3	0–5 b	0.9 (0.2)	2048	3	1–5 c	1.1 (0.2)

^1^ Median lethal time. ^2^ Fiducial limits. Different lowercase letters indicate significant differences in the responses (*p* ≤ 0.05). “-” means bioassay for LT_50_ was not performed.

**Table 6:** Median lethal time (LT_50_) of pyrethroids and organophosphates in female house flies.

Population	Conc. _ppm_	^1^ LT_50_ (h)	^2^ FL (95%)	Slope (SE)	Conc. _ppm_	^1^ LT_50_ (h)	^2^ FL (95%)	Slope (SE)
		Alpha-cypermethrin				Deltamethrin		
Dirab	256	10	5–13 bc	3.6 (1.0)	2048	4	2–8 c	1.1 (0.2)
Al-Masanie	256	13	5–42 abc	0.6 (0.2)	2048	4	1–7 c	1.0 (0.2)
Al-Uraija	256	30	18–71 a	1.1 (0.3)	2048	17	11–26 ab	1.4 (0.3)
Al-Washlah	256	19	13–25 ab	2.2 (0.5)	2048	24	18–35 a	1.5 (0.2)
Al-Muzahmiya	256	3	0–7 c	0.6 (0.2)	2048	6	2–15 bc	0.7 (0.2)
		Bifenthrin				Cypermethrin		
Dirab	2048	5	3–9 b	1.3 (0.2)	2048	1	0–4 a	0.5 (0.2)
Al-Masanie	2048	49	24–262 a	0.8 (0.2)	2048	4	1–7 a	0.9 (0.2)
Al-Uraija	2048	-	-	-	2048	3	1–8 a	0.8 (0.4)
Al-Washlah	2048	14	8–27 ab	1.0 (0.2)	2048	4	1–11 a	0.26 (0.2)
Al-Muzahmiya	2048	17	10–31 a	1.1 (0.2)	2048	1	0.1–3 a	0.7 (0.2)
		Cyfluthrin				Fenitrothion		
Dirab	2048	4	2–7 a	1.4 (0.2)	2048	36	20–113 a	0.9 (0.2)
Al-Masanie	2048	2	0–5 a	0.7 (0.2)	2048	22	18–27 a	3.5 (0.7)
Al-Uraija	2048	2	0–4 a	0.9 (0.2)	2048	14	8–23 a	1.2 (0.2)
Al-Washlah	2048	7	0–31 a	0.4 (0.2)	2048	20	15–25 a	3.1 (0.6)
Al-Muzahmiya	2048	6	1–13 a	0.7 (0.2)	2048	24	17–35 a	1.9 (0.4)
		Chlorpyrifos				Malathion		
Dirab	2048	10	7–14 b	2.0 (0.4)	2048	8	5–11 c	2.3 (0.4)
Al-Masanie	2048	8	5–11 b	2.8 (0.6)	-	-	-	-
Al-Uraija	2048	21	16–27 a	2.6 (0.6)	2048	15	10–21 bc	1.8 (0.3)
Al-Washlah	2048	3	2–4 c	1.9 (0.3)	2048	22	16–31 ab	2.2 (0.5)
Al-Muzahmiya	2048	14	9–21 ab	1.6 (0.3)	2048	34	27–48 a	2.8 (0.6)

^1^ Median lethal time. ^2^ Fiducial limits. Different lowercase letters indicate significant differences in the responses (*p* ≤ 0.05). “-” means bioassay for LT_50_ was not performed.

## Data Availability

The data presented in this study are available from the corresponding author on a reasonable request.
